# Potential of Photodynamic Therapy Based on Sugar-Conjugated Photosensitizers

**DOI:** 10.3390/jcm10040841

**Published:** 2021-02-18

**Authors:** Hiromi Kataoka, Hirotada Nishie, Mamoru Tanaka, Makiko Sasaki, Akihiro Nomoto, Tomohiro Osaki, Yoshiharu Okamoto, Shigenobu Yano

**Affiliations:** 1 Department of Gastroenterology and Metabolism, Nagoya City University Graduate School of Medical Sciences, Nagoya 467-8601, Japan; nishix589998@gmail.com (H.N.); mtanaka77@gmail.com (M.T.); sasakimakiko@gmail.com (M.S.); 2 Department of Applied Chemistry, Graduate School of Engineering, Osaka Prefecture University, Osaka 599-8531, Japan; nomoto@chem.osakafu-u.ac.jp; 3 Joint Department of Veterinary Clinical Medicine, Faculty of Agriculture, Tottori University, Tottori 680-8553, Japan; tosaki@muses.tottori-u.ac.jp (T.O.); yokamoto@muses.tottori-u.ac.jp (Y.O.); 4 KYOUSEI Science Center for Life and Nature, Nara Women’s University, Kitauoyahigashi-machi, Nara 630-8506, Japan; yano-s@cc.nara-wu.ac.jp

**Keywords:** photodynamic therapy (PDT), photosensitizer, chlorin, tumor-associated macrophage (TAM), Warburg effect

## Abstract

In 2015, the Japanese health insurance approved the use of a second-generation photodynamic therapy (PDT) using talaporfin sodium (TS); however, its cancer cell selectivity and antitumor effects of TS PDT are not comprehensive. The Warburg effect describes the elevated rate of glycolysis in cancer cells, despite the presence of sufficient oxygen. Because cancer cells absorb considerable amounts of glucose, they are visible using positron emission tomography (PET). We developed a third-generation PDT based on the Warburg effect by synthesizing novel photosensitizers (PSs) in the form of sugar-conjugated chlorins. Glucose-conjugated (tetrafluorophenyl) chlorin (G-chlorin) PDT revealed significantly stronger antitumor effects than TS PDT and induced immunogenic cell death (ICD). ICD induced by PDT enhances cancer immunity, and a combination therapy of PDT and immune checkpoint blockers is expected to synergize antitumor effects. Mannose-conjugated (tetrafluorophenyl) chlorin (M-chlorin) PDT, which targets cancer cells and tumor-associated macrophages (TAMs), also shows strong antitumor effects. Finally, we synthesized a glucose-conjugated chlorin e6 (SC-N003HP) that showed 10,000–50,000 times stronger antitumor effects than TS (IC_50_) in vitro, and it was rapidly metabolized and excreted. In this review, we discuss the potential and the future of next-generation cancer cell-selective PDT and describe three types of sugar-conjugated PSs expected to be clinically developed in the future.

## 1. Introduction

Photodynamic therapy (PDT) is a mildly invasive anticancer therapy that combines a photosensitizer (PS) and a specific wavelength of light irradiation to generate reactive oxygen species (ROS) to destroy cancer cells. Irradiation light therapy has been used for thousands of years in civilizations as diverse as those of ancient Egypt, India, and China against conditions, such as psoriasis, vitiligo, and skin cancer [1,2,3]. In the past century, several scientists also found that a combination of light and certain chemicals could induce cell death. The modern era of PDT began in the 1960s when Rich Lipson of Mayo Clinic started studying the use of a hematoporphyrin derivative (HPD) [4,5]. PSs, such as HPD, tend to accumulate in tumor tissue, although why this occurs is not fully understood. One theory is that the high vascular permeability of HPD derivatives and their affinity for proliferating endothelium, along with the lack of lymphatic drainage in tumor tissue, might be the cause [6].

To intensify the anticancer effect of PDT and decrease the invasiveness to peripheral normal tissue, increasing the cancer cell selectivity accumulation of the PS and making it better at tumor targeting are very important. Another crucial part of killing cancer cells is the productivity of PDT in creating ROS. Longer wavelength light irradiation is also very important for cancer cell destruction as many cancers exist in the deeper layers of the skin, away from surface light irradiation. The wavelength of the light depends on the characteristic of the PS used. 

Tetrapyrrole structures are a major group of PS used against cancer, alongside PDT. These structures are found in several common molecules such as hemoglobin, chlorophyll, and bacteriochlorophyll. HPD and porphyrins (specifically Photofrin) were the first PSs used in a clinical setting worldwide [7,8]. The Q-band of tetrapyrrole PSs is around 630 nm (porphyrins: 633 nm, chlorins: 650 nm, BODIPY: 523 nm (in ethanol), H_2_-phthalocyanines: 680–700 nm in DMF, Zn-phthalocyanines: 702 nm in DMF).

Chlorins and chlorin e6 structures are also used as PSs, and some of their clinically used forms include m-tetrahydroxyphenylchlorin (temoporfin or Foscan) [9], a benzoporphyrin derivative (verteporfin) [10], and Radachlorin (or Bremachlorin) [11]. Chlorin e6 is derived from naturally occurring chlorophyll, and both chlorin and chlorin e6 are activated by red light between 650 and 700 nm. Bacteriochlorin-derived PSs are also used, such as Tookad [12], as well as its water-soluble derivative known as Tookad Soluble [13]. Near-infrared (NIR) light, between 700 and 800 nm, is used to activate bacteriochlorins. 

To increase the cancer cell selectivity and PS accumulation, we developed PDT using sugar-conjugated PSs. We synthesized more than 30 kinds of sugar-conjugated chlorins and investigated the usefulness of each. Recently, immunocheckpoint inhibitors, such as anti-PD-1/anti-PD-L1 antibodies, were used in a clinical setting for several cancers [14,15,16], and a few basic studies reported the synergistic effects of PDT and immunocheckpoint inhibitors [17,18].

In this review, we discuss the study of PDT with sugar-conjugated chlorins and the future direction of PDT to include collaboration with cancer immunotherapies.

## 2. The Warburg Effect and Glucose-Conjugated (Tetrafluorophenyl) Chlorin

**Figure 1:**
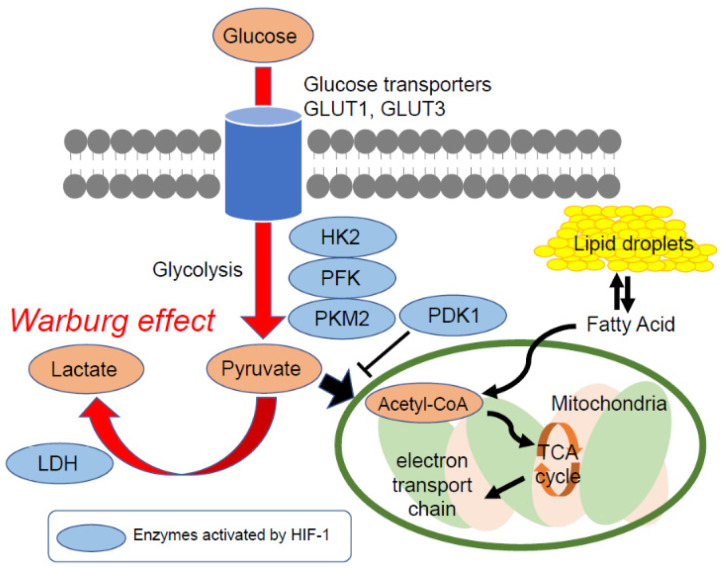
The Warburg effect and typical glucose metabolism in cancer cells. Cancer cells absorb higher levels of glucose than normal cells and generate energy by the non-oxidative breakdown of glucose (glycolysis). Cancer cells are generally found under hypoxic conditions, and glucose uptake and glycolytic pathway metabolisms are upregulated by hypoxia-inducible factor-1 (HIF-1). HIF-1 upregulates glucose transporter expression, such as GLUT1 and GLUT3, and increases glucose uptake. It also activates enzymes for glycolysis such as HK2, PFK, PKM2, and LDH. Lastly, HIF-1 inhibits the pathway at the pyruvate conversion to acetyl-CoA step by activating PDK1.

In the early 19th century, Otto Heinrich Warburg and his colleagues observed that cancer cells absorb significantly more glucose than the surrounding normal cells [19,20]. This is because cancer cells create most of their energy through the anaerobic glycolysis of glucose to produce lactate. Later, this phenomenon became known as the Warburg effect (Figure 1). 

The Warburg effect formed the basis for the development of radioactive glucose analog ^18^F-fluorodeoxyglucose (^18^F-FDG) as a cancer imaging agent for positron emission tomography and computed tomography (PET/CT) scanning [21]. Glucose transporter 1 (GLUT1), a hypoxia-responsive transporter, plays the predominant role in a cancer cell’s uptake of ^18^F-FDG [22]. GLUT1 overexpression has been observed in a broad spectrum of cancers, and GLUT, especially GLUT1, expression is correlated to unfavorable outcomes in patients with cancer [23].

Considering these points, we utilized our understanding of the Warburg effect to develop a third-generation PS as part of a novel PDT, which possesses cancer cell specificity and selectivity. 

**Figure 2:**
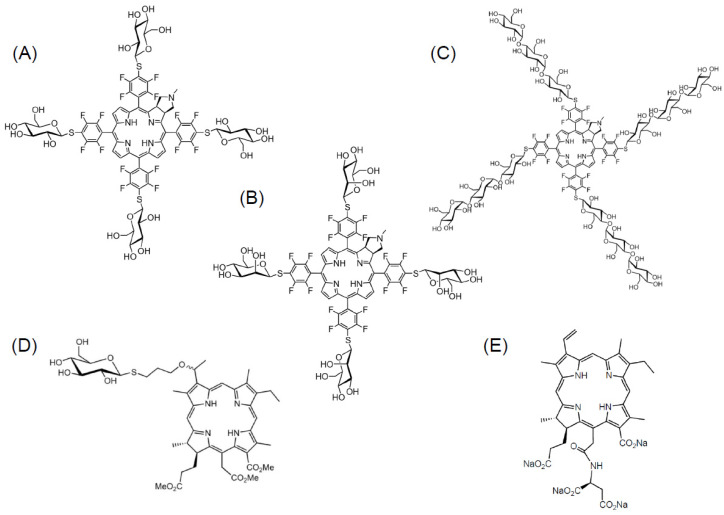
The molecular structure of sugar-conjugated chlorins. (**A**) Glucose-conjugated chlorin (G-chlorin), (**B**) mannose-conjugated chlorin (M-chlorin), (**C**) maltotriose-conjugated chlorin (Mal_3_-chlorin), (**D**) glucose-conjugated chlorin e6 (SC-N003HP), and (**E**) talaporfin sodium. Talaporfin sodium is now clinically used as a second-generation photosensitizer (PS) in Japan.

First, we synthesized glucose-conjugated (tetrafluorophenyl) chlorin (G-chlorin), which acts as the PS. It is a chlorin that is conjugated to four molecules of glucose, thus targeting the GLUT1 of cancer cells [24,25] (Figure 2A). In the in vivo mouse xenograft model, G-chlorin accumulation in a tumor was at its highest four hours after intravenous administration via the tail vein. In vitro IC_50_, G-chlorin showed strong antitumor effects against gastric cancer and colon cancer, and was about 30 times more cytotoxic than talaporfin sodium (TS) PDT [26]. In melanoma-bearing mice, intratumoral G-chlorin injection followed by irradiation with LED light four hours later, controlled tumor growth for 13 consecutive days [27]. 

Gastrointestinal stromal tumors (GISTs) are the most common submucosal mesenchymal tumors in the GI tract [28]. No effective treatment strategies have yet been established for GIST, except surgical resection. GIST positivity is usually determined by a PET scan using fluorine-18-fluorodeoxyglucose (^18^FDG), as GIST cells readily absorb glucose [29]. It is also known that the longer wavelengths of the light spectrum (red, 630–670 nm) can penetrate into the deep layers of the stomach wall. From this information, we speculated that G-chlorin PDT could produce antitumor effects against GIST. We used the GIST cell line and GIST-T1 cells to confirm that G-chlorin PDT shows strong antitumor effects against GIST in a xenograft model [30].

## 3. PDT Enhances Tumor Immunity

PDT is known to induce cancer cell death through three mechanisms: (1) destruction of cancer cells by inducing ROS, in particular singlet oxygen (^1^O_2_); (2) cancer tissue ischemia, where PDT shuts down tumor blood vessels; and (3) stimulation of the host immune system by increasing cancer cell-derived antigen presentation to T cells [31,32]. 

It has been proposed that the damage-associated molecular patterns produced by dying cancer cells play an important role in inducing immunogenic cell death (ICD) [33]. In this process, the immunogenic factor calreticulin (CRT) is translocated from the endoplasmic reticulum to the cell surface by PDT. CRT on the cancer cell surface acts as an “eat me” signal and promotes phagocytic uptake of cancer cells by dendritic cells (or antigen presentation cells). PDT also releases high mobility group box 1 (HMGB1), a ligand for toll-like receptor 4, from the dying cells to the extracellular space. HMGB1 interacts with several receptors expressed on the surface of dendritic cells, including toll-like receptor 4, and activates them [34,35].

G-chlorin PDT induces translocation of CRT and HMGB1 from the nucleus to the cytosol and cell surface of cancer cells. We also confirmed that a vaccine effect results from injecting cancer cells with G-chlorin PDT and that this vaccine effect is cancelled by silencing CRT and HMGB1 with small-interfering RNAs [36].

**Figure 3:**
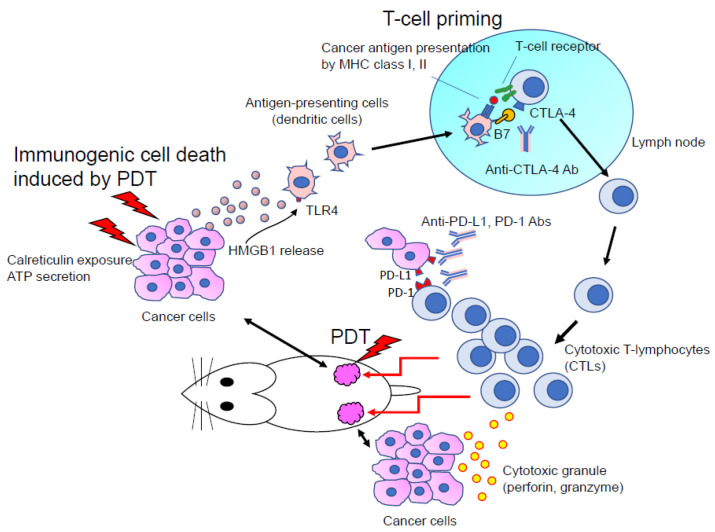
The synergistic effect of photodynamic therapy (PDT) and immune checkpoint blockade for anticancer effects. Immunogenic cell death (ICD) induced by PDT enhances the cancer antigen presentation by dendritic cells (DCs) through DC phagocytosis via calreticulin exposure, adenoScheme 1. (HMGB1) release. The enhancement of T cell priming results in antitumor effects facilitated by cytotoxic T lymphocytes (CTLs). Immune checkpoint blockade (anti-CTLA-4, anti-PD-1, anti-PD-L1 antibodies) synergistically enhance T cell priming and CTL’s attack against cancer cells.

Recently, several studies have confirmed the collaborative effects of ICD induced by PDT and checkpoint blockade cancer immunotherapy with PD-1/PD-L1 antibodies [17,18] (Figure 3). PDT promotes dendritic cells’ phagocytic uptake of dying cancer cells by upregulating CRT and HMGB1. This step consequently enhances the presentation of cancer antigens to T cells through MHC class I and II proteins on dendritic cells, thus enhancing the priming and activation of T cells [37]. This is also how the checkpoint blockade cancer immunotherapy using PD-1/PD-L1 antibodies works; it enhances the attacking and killing of cancer cells by priming and activating T cells (CD8 + T cells). 

From the viewpoint of the cancer immunity cycle, the synergy of the antitumor effects of PDT combination therapy, inducing CRT and HMGB1, and of checkpoint blockade cancer immunotherapy, using PD-1/PD-L1 antibodies, is strongly expected. Radiation therapy against cancer also possesses ICD effects. Several phase II and III clinical studies combining radiation therapy with checkpoint blockade cancer immunotherapy using PD-1/PD-L1 antibodies are now ongoing [38,39]. The clinical study of PDT combination therapy and checkpoint blockade cancer immunotherapy is also soon expected.

## 4. Targeting Tumor-Associated Macrophages in the Tumor Microenvironment

**Figure 4:**
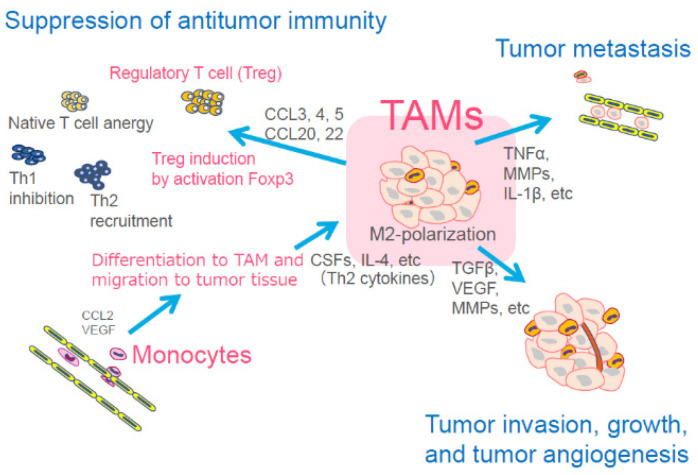
Main function of tumor-associated macrophages (TAMs) for cancer cells. TAMs are differentiated from monocytes and migrate to the tumor tissue. TAMs then accelerate tumor metastasis, tumor invasion, tumor growth, and angiogenesis. TAMs also induce suppression of antitumor immunity through induction and activation of regulatory T cells (Treg). CCL: chemokine (C-C motif) ligand, TNF: tumor necrosis factor, MMPs: matrix metalloproteinase, IL: interleukin, CSFs: colony-stimulating factors, TGFβ: transforming growth factor beta, VEGF: vascular endothelial growth factor.

Cancer cells possess the ability to evade immune response, thus promoting tumor growth and metastatic potential in the tumor microenvironment, which creates hypoxia. The tumor microenvironment is composed of endothelial cells, fibroblasts, immune cells, and various chemokines and growth factors [40,41]. From the different members of the tumor microenvironment, we focused on the specific immune cells of tissue macrophages, which are key regulators of the relationship between the immune system and the tumor. There are two tissue macrophage phenotypes: the classic M1 and the alternative M2. Tumor-associated macrophages (TAMs), which have an M2 phenotype, are implicated in tumor promotion, metastasis, neovascularization, and immunosuppression by activating regulatory T cells (Tregs) and suppressing cytotoxic T lymphocytes (CTLs). TAMs are also associated with poor response to cancer therapy [42,43,44] (Figure 4). A number of clinical studies have also demonstrated that a high infiltration of TAMs into cancer stroma correlated to a poor prognosis in several cancer types [45,46,47]. With this information, we realized that targeting TAMs is a new therapeutic approach for cancer. 

Several TAM-targeting therapies have been observed. For instance, CSF1 (M-CSF)/CSF1R signaling is a key regulator of TAM differentiation and survival [48,49]; therefore, blocking the CSF1/CSF1R pathway is considered to be effective for targeting TAMs. 

Monoclonal antibody (RG7155) that blocks the CSF-1 receptor (CSF1R) dimerization depleted TAMs from the tumor tissue with an increase of the CD8+/CD4+T cell ratio in an animal model. A phase I clinical trial of RG7155 treatment involving patients with diffuse-type giant cell tumors revealed partial responses for five of the seven patients (71%) with the decrease of TAMs in tumor tissue [50]. In the tumor microenvironment, carcinoma-associated fibroblasts (CAF) are the major sources of chemokines that recruit granulocytes to tumors [51]. CSF1 produced by tumor cells inhibits the production of granulocytic chemokines by CAFs. Thus, CSF1 limits granulocyte recruitment to the tumor tissue. Inhibition of CSF1R reversed this effect and induced accumulation of tumor-promoting polymorpho-nuclear myeloid-derived suppressor cells (PMN-MDSC). Therefore, the combination of the CSF1R inhibitor with the CXCR2 inhibitor prevents PMN-MDSC migration. Kumar et al. reported that combining CSF1R and CXCR2 inhibitors reduced the infiltration of both TAM and PMN-MDSC in the tumor tissue and significantly reduced tumor growth [52]. 

Bisphosphonates (BPs) are antiresorptive agents that are already approved for the treatment of skeletal complications associated with metastatic breast and prostate cancer. Recently, BPs, such as zoledronic acid, were reported to suppress TAM differentiation and induce a change of the TAM’s phenotype from pro-tumoral M2 to tumoricidal M1 [53,54].

TAMs express mannose receptors (CD206) on their cell membrane [55]; therefore, we synthesized mannose-conjugated (tetrafluorophenyl) chlorin (M-chlorin) to target the mannose receptors on TAMs (Figure 2B). In this study, we established M1-polarized macrophages and TAM-like M2-polarized macrophages from the THP-1 human monocyte cell line. Then, we confirmed that M-chlorin PDT did not induce cell death in M1-polarized macrophages, but it strongly induced cell death in M2-polarized macrophages like TAMs. In an in vivo syngeneic model study, M-chlorin PDT significantly decreased the number of TAMs in cancer tissue and showed strong antitumor effects. M-chlorin PDT also killed cancer cells because GLUT1 on their surface can incorporate both mannose and glucose [56]. The development of a new PDT, targeting other members of the tumor microenvironment, such as Tregs, cancer-associated fibroblasts (CAFs), neovascular endothelial cells, or cancer cells, is expected to increase antitumor effects.

## 5. Water-Soluble Oligosaccharide-Conjugated (Tetrafluorophenyl) Chlorin 

Water solubility is very important when considering the clinical use of PSs for PDT. To improve the water solubility of sugar-conjugated chlorins, we synthesized a maltotriose-conjugated (tetrafluorophenyl) chlorin (Mal_3_-chlorin) [57] (Figure 2C). Mal_3_-chlorin is a hydrophilic PS and has shown excellent tumor accumulation as a bifunctional PS for PDT and for photodynamic diagnosis (PDD) [58,59]. 

Japanese health insurance has also approved 5-aminolevulinic acid (5-ALA) for brain tumor (malignant glioma) and bladder tumor PDD [60,61]. 5-ALA is transported into cancer and normal cells through oligopeptide transporters 1 or 2 [62,63]. 5-ALA is then metabolized into the active compound protoporphyrin IX and emits strong red fluorescence with excitation of blue-violet light [64]. Because protoporphyrin IX preferentially accumulates in cancer cells, the red fluorescence it emits is a good hallmark for cancer detection. In the cases of malignant glioma, approximately 80%–90% of tumors show red fluorescence in surgery [65]. 

Using MKN 45 human gastric cancer cell lines in vitro, we confirmed that PDD with Mal_3_-chlorin emitted a much stronger red fluorescence (635–650 nm) than that of 5-ALA by irradiation blue light (405–420 nm). PDD with Mal_3_-chlorin also showed much stronger red fluorescence (about 10 to 30 times) than 5-ALA in a xenograft model. From these observations, we consider the Mal_3_-chlorin as a water-soluble bifunctional PS for PDD and PDT.

## 6. Other Expected Photosensitizers

There are three other types of PSs (fullerene, phthalocyanine (Pc), and indocyanine green (ICG)) with sugar conjugation capabilities that are expected to be clinically developed in the future. Because these compounds have long wavelength electron absorption peaks along with excellent tissue permeability, they are important for the further clinical development of PDT. 

### 6.1. Fullerenes (C60, C70)

**Figure 5:**
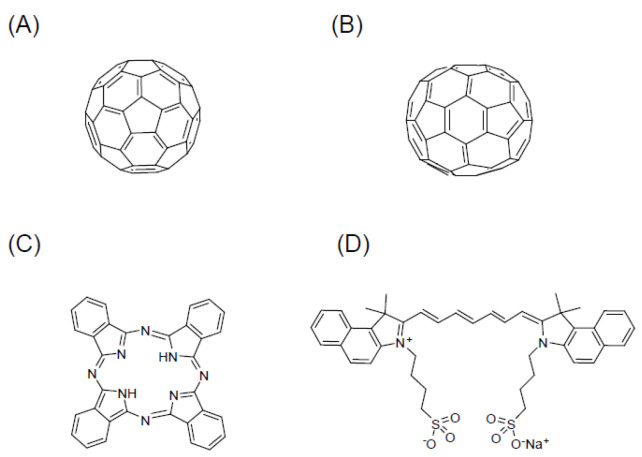
The molecular structure of types of PSs photosensitizers that are expected to be clinically developed in the future due to sugar conjugation. (**A**) Fullerene (C60), (**B**) fullerene (C70), (**C**) phthalocyanine (Pc), and (**D**) indocyanine green (ICG).

Fullerenes (C60, C70) are considered a new class of carbon molecules. Fullerene, discovered in 1985, is composed of 60 carbon atoms arranged in a soccer ball-like structure (Figure 5A) [66]. The first photophysical study of fullerenes, reported by Foote and his coworkers, described the appearance of an excited state of C60 (^1^C60* and ^3^C60*) that subsequently afforded ^1^O_2_ through an energy transfer mechanism [67]. Fullerenes (C60, C70) (Figure 5B) are particularly useful due to their long wavelength of absorption (S-S absorption: 530, 920 nm (C60); 570, ~740, ~900 nm (C70)), high quantum yield to form ROS (1.0 based on generation of singlet oxygen, ^1^O_2_), and lack of acute toxicity in the absence of light, except in rare cases [68,69]. Fullerenes, however, are known to have the drawback of a poor solubility and no tumor selectivity. Sugar conjugation with C60 would allow the introduction of both hydrophilicity and tumor selectivity to a fullerene. PDT using sugar-conjugated C60 derivatives has been reported [70,71,72,73]. Sugar coating of C70 can be expected to lead to the development of useful PSs as well [74].

### 6.2. Phthalocyanine

Pc is a cyclic compound with a structure in which four phthalimides are bridged by nitrogen atoms (Figure 5C). Its structure is similar to porphyrin. Pc is an important substance class for both classical and modern applications like fluorescence probes and for medical applications, such as photochemical internalization and PDT. Pcs are relevant for clinical use mainly due to their strong absorption in the far-red spectral band (λ-670 nm), but only if strong dye lasers are available and if the tissue is more transparent than ~630 nm. Pc has serious limitations concerning biocompatibility and biosolubility; however, it has so far required special chemical formulations to be used in biomedical applications.

To overcome these limitations, Pc cores have been chemically modified by decoration with biocompatible motifs such as sugars [75,76,77]. The C-glycosidically substituted Pcs did not form any aggregation in solution. Therefore, they were considered as potential PSs in PDT [78]. As a result, it is expected that the conjugation of sugar and Pc will lead to the development of innovative drugs for PDT and PDD. A review focused on the latest technology in the development of drug delivery systems, using Pc as a PS for PDT, has been reported [79]. 

### 6.3. Indocyanine Green 

ICG (Figure 5D) has the attractive features of very low toxicity and high absorbance in wavelengths ranging from 600 to 900 nm. This is a relatively transparent window for biological tissue. ICG is a suitable NIR light-absorbing compound and is used in diagnostic and medical applications, as well as for the treatment of cancer by laser-mediated photothermal therapy [80], and theranostics [81,82,83]. However, the application of ICG for clinical imaging and therapeutic applications has several limitations, such as a short plasma half-life and poor aqueous stability. Various trials including nanoformulations of ICG [84,85] or sugar conjugation of ICG [86,87,88] have been developed to overcome these limitations. 

## 7. Preclinical Studies of a Glucose-Conjugated Chlorin e6 (SC-N003HP)

We developed a glucose-conjugated chlorin e6 (SC-N003HP) (Figure 2D). SC-N003HP showed the best antitumor effects and was approximately 10,000–50,000 times stronger than TS (IC_50_) in vitro [89]. Figure 2 shows the chemical structure of all the sugar-conjugated chlorins mentioned in this paper. SC-N003HP had the strongest antitumor effects in vitro among the 30 kinds of sugar-conjugated chlorins we synthesized. In syngeneic in vivo models, PDT and SC-N003HP also revealed strong antitumor effects with a 40% complete response rate.

Because we are aiming for clinical use, we performed an absorption, distribution, metabolism, and excretion preclinical test of SC-N003HP using ^14^C-labeled SC-N003HP. In a rat model, we discovered that the half-life in blood was 1.8 days and that 94.9% of SC-N003HP was excreted into bile after 24 h. The minimum lethal dose was 250 mg/kg in mice and 300 mg/kg in rats. These results were superior to those of TS. 

**Figure 6:**
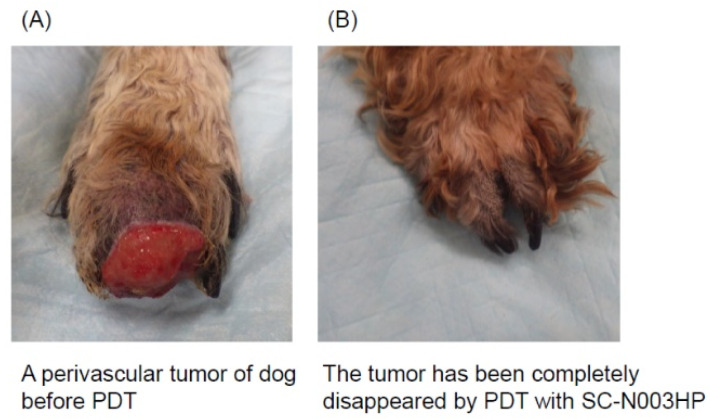
A case of dog with a perivascular tumor on the foot (stage T2N0M0) cured by PDT with SC-N003HP. (**A**) The perivascular tumor on the dog’s foot before PDT; (**B**) the tumor completely disappeared using PDT with SC-N003HP.

Finally, we examined the effects of PDT with SC-N003HP using the case of a dog with a spontaneous tumor. The PDT protocol was approved by the Ethics Committee at the Faculty of Agriculture, Tottori University (ethical approval number: H28-004). The dog (male, 14 years old) had a perivascular tumor on the foot (stage T2N0M0) (Figure 6). We intravenously administered 2 mg/kg SC-N003HP and irradiated with a semiconductor laser (wavelength: 671 nm, 160 mW/cm, 80 J/cm). PDT was performed a total of six times, and 103 days later the tumor completely disappeared. This preclinical trial of a dog with a foot tumor was very important, and from the trial we obtained evidence of SC-N003HP and PDT’s antitumor effects and safety.

In conclusion, SC-N003HP, with its strong antitumor effects and improved safety, is regarded as the best candidate for a next-generation PDT among the sugar-conjugated chlorins we synthesized.

## 8. Summary and Perspectives

Reports on many types of nanoparticles, including PSs, have recently been published [90]. Specific antibodies against cancer cell surface molecules conjugated to PSs are being developed for use with NIR irradiation (photoimmunotherapy) [91,92,93]. Clinical studies are also underway for photoimmunotherapy in patients with recurrent head and neck cancer using the photoabsorber IR700 conjugated to cetuximab (Erbitux^®^), an epidermal growth factor receptor antibody. Novel PDT, when used with an antibody-conjugated photosensitizer (PS), is one of the newest strategies for cancer cell-selective PDT. Heterogeneity of the target molecule’s expression in cancer cells is a problem, particularly to achieve complete remission in human cancers. Conjugating PSs with peptides that target molecules on the cancer cell surface is another strategy. PS conjugated with an RGD peptide targeting the α_v_β_3_ integrin, which is overexpressed in tumor cells and tumor-associated blood vessels, showed specific accumulation in tumor tissue and tumor blood vessels [94,95]. The A20FMDV2 peptide, originated from the VP1 coat protein of the foot-and-mouth disease virus, and the HK peptide specifically target the α_v_β_6_ integrin on expressing tumors [18,96,97,98].

As mentioned before, targeting the members that comprise the tumor microenvironment (e.g., TAMs) is another strategy. The RGD peptide for the α_v_β_3_ integrin can target tumor-associated micro-blood vessels. The organic multimodal phototheranostic nanosystems of activated fibroblasts semiconducting polymer nanoparticles displayed specific targeting of CAFs [99]. TAMs are known to enhance the recruitment of Tregs by secreting TGF-β and CCL2, 3, 4, 5, and 20. Thus, TAM depletion by M-chlorin PDT indirectly inhibits Treg recruitment [42].

Cancer immunotherapy, particularly the method of immune checkpoint blockade, has received more interest because of its potential clinical antitumor effects. However, the response rate has been insufficient, only about 10%–15% in gastrointestinal cancer. Further improvements in therapeutic effects are expected, and combining this therapy with other treatments may help obtain a synergistic therapeutic outcome. There have been several studies focusing on PDT-synergized cancer immunotherapies [18,100,101,102]. One PDT and cancer immunotherapy combination study revealed that CD8^+^ T cells can infiltrate distant tumors and primary irradiated tumors, with strong tumor killing activity [17]. These effects are thought to occur through the synergy of enhancing both cancer antigen presentation and T cell priming via ICD induction using PDT and enhancing T cell priming, recognition, and killing of cancer cells via immune checkpoint blockade (anti-PD-1 antibody).

In conclusion, we discussed the development of sugar-conjugated chlorins and chlorin e6 including preclinical studies using a dog in our group. New findings of tumor microenvironment, cancer immunology, drug delivery systems, and induction of immunogenic cell death by PDT may advance future development of PDT. We hope that this review plays a role for the development of next-generation PDT as one of the effective cancer therapies.

## Data Availability

Not applicable.
